# Oil Production by Pyrolysis of Real Plastic Waste

**DOI:** 10.3390/polym14030553

**Published:** 2022-01-29

**Authors:** Laura Fulgencio-Medrano, Sara García-Fernández, Asier Asueta, Alexander Lopez-Urionabarrenechea, Borja B. Perez-Martinez, José María Arandes

**Affiliations:** 1 Gaiker Technology Center, Basque Research and Technology Alliance (BRTA), Parque Tecnológico de Bizkaia, Edificio 202, 48170 Zamudio, Spain; fulgencio@gaiker.es (L.F.-M.); garciasa@gaiker.es (S.G.-F.); asueta@gaiker.es (A.A.); 2 Chemical and Environmental Engineering Department, Faculty of Engineering of Bilbao, University of the Basque Country (UPV/EHU), Plaza Ingeniero Torres Quevedo 1, 48013 Bilbao, Spain; borjabperez@gmail.com; 3 Department of Chemical Engineering, University of the Basque Country (UPV/EHU), P.O. Box 644, 48080 Bilbao, Spain; josemaria.arandes@ehu.eus

**Keywords:** chemical recycling, plastic waste, industrial rejected streams, pyrolysis oil, pyrolysis, secondary raw materials, alternative fuels

## Abstract

The aim of this paper is for the production of oils processed in refineries to come from the pyrolysis of real waste from the high plastic content rejected by the recycling industry of the Basque Country (Spain). Concretely, the rejected waste streams were collected from (1) a light packaging waste sorting plant, (2) the paper recycling industry, and (3) a waste treatment plant of electrical and electronic equipment (WEEE). The influence of pre-treatments (mechanical separation operations) and temperature on the yield and quality of the liquid fraction were evaluated. In order to study the pre-treatment effect, the samples were pyrolyzed at 460 °C for 1 h. As pre-treatments concentrate on the suitable fraction for pyrolysis and reduce the undesirable materials (metals, PVC, PET, inorganics, cellulosic materials), they improve the yield to liquid products and considerably reduce the halogen content. The sample with the highest polyolefin content achieved the highest liquid yield (70.6 wt.% at 460 °C) and the lowest chlorine content (160 ppm) among the investigated samples and, therefore, was the most suitable liquid to use as refinery feedstock. The effect of temperature on the pyrolysis of this sample was studied in the range of 430–490 °C. As the temperature increased the liquid yield increased and solid yield decreased, indicating that the conversion was maximized. At 490 °C, the pyrolysis oil with the highest calorific value (44.3 MJ kg^−1^) and paraffinic content (65% area), the lowest chlorine content (128 ppm) and more than 50 wt.% of diesel was obtained.

## 1. Introduction

Nowadays, the huge growth in plastic production has resulted in a massive generation of this kind of waste. Despite not being considered hazardous waste, plastic waste causes cumulative and long-term environmental impacts due to its long lifespan [1,2]. In order to reduce adverse effects presented by plastic waste, a recent European Directive 2018/851 was renewed to promote the recovery of plastic waste for recycling, avoiding the deposition in landfills [3]. Nevertheless, the amount of waste that ends up in landfills is still very high. According to a recently published report, in Spain, landfill is the most recurrent measure to get rid of post-consume plastic waste (46%) [4]. Increasing the recycling rate and reducing the landfill disposal only through conventional mechanical recycling routes is sometimes complicated and not an economically viable alternative, since there are a lot of plastic waste streams that are composed by a wide and intermingled variety of materials, especially those that came from industrial recovery processes [5,6]. Therefore, new recycling alternatives are required, and pyrolysis, recently catalogued as TRL 9 (technology readiness level), seems to be a promising option [7].

The pyrolysis process consists of the thermal degradation of organic materials under an inert atmosphere. During the pyrolysis of plastics, the long carbon chains are thermally broken down into useful fractions that can serve as fuels or sources of chemicals. Typically, a liquid product, a gaseous product and a solid product are formed [8]. The solid product is usually made up of the inorganic elements of the waste (including the charges in plastics), together with the so-called “char”, a carbonaceous product typical of the thermal decomposition of some polymers. Due to its heterogeneity, the solid product is not usually easy to valorise. On the contrary, the gaseous fraction normally meets the standards of a gaseous fuel, but its economic value is not sufficient to be the exclusive product of the process. Consequently, the economic success of the pyrolysis of plastic waste depends on the characteristics of the liquid product, which in principle can be largely assimilated to certain refinery streams [9]. In fact, given the petrochemical origin of plastics, returning them to the refineries when they have reached the end of life should be their circular route, provided that they cannot be mechanically recycled. In such a scenario, the pyrolysis of plastic waste allows for two benefits: the reduction of landfill disposal and the recovery of valuable hydrocarbons [10].

The characteristics and yields of the products depend to a great extent on various parameters of the pyrolysis process: temperature, residence time, reactor type, pressure, type and rate of fluidizing gas, heating rate, type of catalyst and type of feedstock [11,12,13]. As pyrolysis is a thermal process, the temperature is the major operational factor since it controls the cracking reactions of the polymer chains. It was reported that temperatures of the 300–500 °C range favoured conversion into liquid products [10,14]. Even though pyrolysis can tolerate mixtures of different types of plastics [5,15], polyolefins have turned out to be the most appropriate, since they produce liquid oils with low octane numbers, which are comparable to conventional fuel [15,16,17]. There are many references in the literature about the pyrolysis of virgin plastic and prepared plastic waste mixtures in order to achieve liquid fuel. However, few authors have analysed the pyrolysis of real waste samples which results in different liquid products in terms of composition and quality, owing to its great complexity [5,18,19]. Some undesirable materials usually present in real waste streams (PVC, metals, PET, inert materials and cellulose-based materials) deteriorate the quality of the pyrolysis products obtained. On the one hand, chlorine from PVC is detrimental since chlorinated compounds can be formed in the liquid product decreasing its quality and limiting its application [5,20,21]. The metals contained in the initial samples might remain unaltered during the pyrolysis process and could be recovered from solid product [20], but it might also produce an undesired catalytic effect [18,22,23,24,25,26] and of course, as part of the solid fraction, they do decrease the yield of liquids and gases. PET and cellulosic materials favour the formation of char and an aqueous phase in the pyrolysis liquid [27,28,29,30]. Thus, the source and the previous treatment of these waste streams influence the properties of the final products. Nonetheless, there are no publications analysing the effect of treatments applied to the waste stream prior to pyrolysis in order to improve the quality of the liquid obtained. Hence, in this study, the waste stream composition to pyrolize is another parameter to be studied.

In this research, three real samples were collected from different plastic-rich waste streams rejected from industrial operations and whose final disposal is normally landfill. These samples were used as feedstock in the pyrolysis process to evaluate the production and quality of the liquid products, in order to be considered for their application in refineries. The samples were processed as received and after using different pre-treatments to separate the non-desired components that could downgrade the pyrolysis oil quality. Once the effect of the pre-treatment was studied, the sample producing the most appropriate pyrolysis oil to be used as feedstock for refineries was selected. This sample was employed to investigate the effect of temperature on the production of pyrolysis oil.

## 2. Materials and Methods

### 2.1. Origin of the Samples

The samples used in this research were provided by three different recycling companies of the Basque Country (Spain). The origin and type of such waste streams, together with the annual amount generated in the Basque Country in 2017 are summarised in Table 1. The first sample was collected from a light packaging waste classification plant; there, the main components of the light packaging selectively collected in Bizkaia (a region of Basque Country) are separated for their subsequent use as raw material in recycling companies. Although more than 70% of the collected packaging waste is properly classified, there is a rejected stream with non-separated materials that is incinerated or deposited in landfill. This sample mainly consisted of PE bags and films caked with dirt, as can be seen in the picture in Table 1. The sample was named “Film sample”. The second sample was collected from a company devoted to the production of newsprint paper from wastepaper recovered in street containers. As a consequence of the separation processes, a plastic containing rejected stream is also generated in this plant, mainly consisting of polyolefins and cellulosic materials. In this case, the sample was named “Paper sample”. The third sample was a rejected stream coming from waste of electrical and electronic equipment (WEEE) and taken from a company devoted to dismantling and shredding WEEE to obtain high-quality metal fractions for its commercialization. This sample was named “WEEE sample”.

Representative samples were obtained by quartering method according to the C702 and D75 ASTM Standards. Afterward, the composition was qualitatively and quantitatively determined. First, a manual separation based on visual identification was carried out in order to separate the materials into macroscopic components (plastics, wood, textiles and inert materials). Next, the specific composition of the plastic fraction was determined by infrared spectroscopy and flame test.

### 2.2. Pre-Treatment Techniques

According to the composition and the specific characteristics of each waste stream, different mechanical separation technologies were applied to reduce the non-desired components in each case. For the Film sample, the separation method used was flotation (sink/float), as it takes advantage of the difference between the density of PVC and the main plastics present in the waste, i.e., polyolefins and styrene polymers (ABS and PS). Paper sample was previously deagglomerated in a jaw shredder (Oliver&Battle SOPAC-100, Badalona, Spain) to improve materials separation. After a previous screening of pre-treatment technologies for the paper-based stream, the optical separation was the selected method, as it showed the highest reduction in PVC concentration. For this purpose, automatic identification and sorting pilot line (UNISORT PX800F, RTT Systemtechnik GmbH, Zittau, Germany), based on a near-infrared (NIR) spectrophotometer (4000–10,000 cm^−1^ spectral range) and an air ejection, was employed. In this equipment, the waste flow placed on a conveyor belt passes under the measuring module (KUSTA 4004M20, LLA Instruments GmbH, Berlin, Germany) and is irradiated with IR light, which is partly absorbed. The reflected light is captured by the sensor and conducted to the spectrophotometer, obtaining the characteristic infrared spectrum of each material. The most suitable technology employed for the pre-treatment of WEEE sample was the densimetric table since it was capable of separating PVC wires from other particles taking advantage of their different morphology. The equipment used (PETKUS KD50, Palencia, Spain) combines the movement of the table with the air-flow generated by the fans, which makes the materials slide on its surface and enables the effective separation of wires, among other undesired elements.

### 2.3. Pyrolysis Experiments

**Figure 1:**
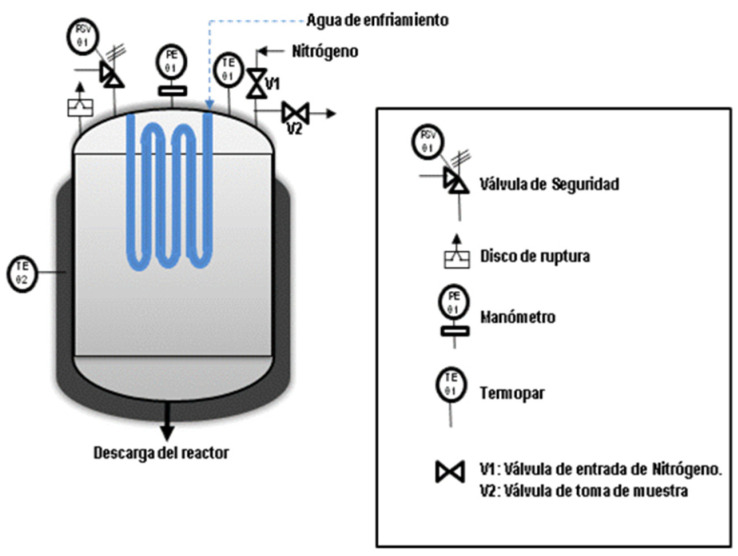
Pyrolysis rector.

For pyrolysis experiments, typically 85 g of crushed samples (dp < 8 mm) were placed in a 2 L unstirred stainless steel autoclave (4570 model of Parr Instruments (Moline, IL, USA), see Figure 1). Prior to the experiments, the system was purged for 20 min with an N_2_ stream, which was kept constant during reaction (80 mL min^−1^). Then, the reactor was heated to the selected experiment temperature (430–490 °C) at a rate of 15 °C min^−1^. As the vapours were generated, they left the reactor passing through a water-refrigerated condenser where the condensable liquids were collected. After an isothermal holding time of 1 h, the reaction system was cooled down to ambient temperature. The solid residue collected inside the reactor and the condensed liquids were weighted, and their yields were calculated according to Equation (1). The gas product yield was calculated by difference.

(1) $$\mathrm{Product}\;\mathrm{yield}\;(\mathrm{wt}.\%)=\frac{M_{\mathrm{product}}(g)}{M_{\mathrm{feed}}(g)}\cdot 100$$

### 2.4. Analytical Techniques

Both raw and pre-treated waste samples were thoroughly characterized using the following analytical techniques. Thermogravimetric analyses (TGA) of the waste samples were carried out in a Mettler-Toledo (Columbus, OH, USA) thermobalance (TGA/DSC1 Stare System). Approximately 10 mg of sample were introduced into the thermobalance and heated to 800 °C at 20 °C min^−1^ rates under a constant N_2_ flow (50 mL min^−1^). The mass loss was continuously monitored as a function of temperature. The derivative thermogravimetric curve (DTG) was calculated to determine the range of temperatures in which the greatest thermal degradation took place. Furthermore, proximate analysis (moisture, volatile matter, fixed carbon, ash) was carried out according to D3173-85 and D3174-82 ASTM standards in LECO TGA-701 equipment (St. Joseph, MI, USA). The C, H, N and S contents (ultimate analysis) were measured by a CHN-S automatic analyser (LECO TrueSpec CHN and TrueSpec S, St. Joseph, MI, USA). The content of chlorine was measured following the UNE 15408 standard, which consists of combusting the samples in a calorimetric bomb (1356 Parr Instrument, Moline, IL, USA) with pure oxygen and absorbing the combustion gases generated in a basic solution of KOH (0.2 M). The concentration of chloride anions present in the solution was then quantified by high-performance liquid chromatography (HPLC) using an ion chromatograph (ICS-1000 DIONEX, Sunnyvale, CA, USA). Waste samples were also digested with H_2_SO_4_ at 180 °C for 15 min and subsequently with an oxidizing mixture of H_2_O_2_ and HNO_3_ at 200 °C for 20 min in order to determine the metal content. Metals were then analysed in aqueous phase by inductively coupled plasma-optical emission spectrometry (ICP-OES) using Optima 2100 DV Perkin-Elmer equipment (Waltham, MA, USA. At last, bulk density of samples was determined by weighting the mass occupied in a measured volume.

Concerning pyrolysis oils, a gas chromatograph (GC 6890N), equipped with an HP-5MS capillary column (30 m × 0.25 mm × 0.25 μm) and coupled to a mass spectrometer detector (MS 5975), both from Agilent Technologies (Santa Clara, CA, USA), was used to determine their composition. The higher heating value (HHV) was measured by using the 1356 Parr calorimetric bomb, which at the same time was also used to determine the halogen content (F^−^, Cl^−^ and Br^−^) following the aforementioned UNE 15408 standard. Metal content of oils was established by digestion with an oxidizing mixture of H_2_O_2_ and HNO_3_ at 200 °C for 20 min followed by ICP-OES. Finally, simulated distillation analyses were carried out according to the ASTM D2887 standard, using an Agilent Technologies 6890 GC System (Santa Clara, CA, USA), equipped with: (i) an FID detector; and (ii) a DB-2887 semi capillary column (length, 10 m; internal diameter, 0.53 mm; thickness, 3 μm).

## 3. Results

### 3.1. Influence of the Pre-Treatment Techniques

#### 3.1.1. Properties of Waste Samples

Table 2 shows the material composition of the collected waste samples, both in the “raw” condition (as received) and after pre-treatment. Bulk density of samples is also included in Table 2, together with two calculated ratios that allow quantifying the effectiveness of the pre-treatment techniques (see Equations (2) and (3)): “total material recovery” (TMR) and “recovery of material suitable for pyrolysis” (RMSP). While TMR refers to the amount of material obtained after pre-treatment (separation of unwanted materials), the term RMSP refers to the concentration of plastics in the recovered fraction that is appropriate for the pyrolysis process in order to obtain high liquid yields. In this research, polyolefins and styrenics were considered as the most suitable plastics for such an objective.

(2) $$\mathrm{TMR}\;(\mathrm{wt}.\%)=\frac{\mathrm{recovered}\;\mathrm{material}\;(\mathrm{kg})}{\mathrm{initial}\;\mathrm{material}\;(\mathrm{kg})}\cdot 100$$

(3) $$\mathrm{RMSP}\;(\mathrm{wt}.\%)=\frac{\mathrm{suitable}\;\mathrm{recovered}\;\mathrm{plastics}\;(\mathrm{kg})}{\mathrm{recovered}\;\mathrm{material}\;(\mathrm{kg})}\cdot 100$$

The raw Film sample is the sample that showed the highest plastic content, mainly composed of polyolefins (75 wt.%). In addition, its high content of PVC, PET and inorganic matter, the last formed by aluminium cans that were trapped inside the PE bags, was remarkable. In the flotation process, most of the polyolefins, whose density is less than 1.0 g cm^−3^, floated to the surface while other polymers such as PVC and PET, and inorganic materials, whose densities are greater, sank to the bottom. Hence, the content of such undesirable components was significantly reduced during the pre-treatment. So, flotation was enabled to recover the 93.0 wt.% of the MSP, mainly formed by polyolefins (increase from 75.0 to 93.1 wt.%) with a high TMR (78.5 wt.%). It is reported that other authors employing flotation methods to separate plastics were used wetting agents in the process. Pongstabodee et al. used 30% *w/v* calcium chloride solution to separate PP and HDPE from a mixed post-consumer plastic waste (PET, PVC, PS and ABS) [31] and Guo et al. employed a solution with 70 mg L^−1^ of sodium dodecyl sulphate to separate PS from a mixture of PET, PVC and PC from light packaging waste [32]. However, in this case, the employment of wetting agents was not necessary to obtain high TMR and RMSP rates.

The raw Paper sample presented an important content of cellulosic materials (31.5 wt.%), principally paper and paperboard, which was expected because of the origin of the sample. Nevertheless, polyolefins constituted again the main fraction (36.1 wt.%). Moreover, it is important to highlight the high content of inorganic materials (13.4 wt.%) as well as the non-desired plastics, PVC (2.8 wt.%) and PET (5.3 wt.%) in the raw sample. In this case, the optical separation equipment achieved the removal of cellulosic material, reducing its content up to 18.3 wt.%. This resulted in a lower concentration of such oxygenated polymers, which might also improve the quality of the oil. It is also remarkable the strong reduction of inorganics (from 13.4 to 2.5 wt.%) and to a lesser extent that of PVC (from 2.8 to 1.5 wt.%) and PET (from 5.3 to 2.7 wt.%). However, compared to the other treatments, this method showed the lowest percentage of RMSP (43.7 wt.%). The separation difficulty of this sample lay in the fact that the paper was very intermingled with plastic and other materials and, in spite of the previous sample deagglomeration, the optical separation equipment could not properly identify and separate the desired polymers. In this case, the incorporation of a previous wet stage with some agent could have resulted in a better separation of polyolefins and paper. In fact, the dissolution of adhesive resins of polyolefins with the aim of separating polyolefins from post-consumer recycled paper was previously reported [33].

The WEEE sample contained plastics of diverse nature, as can be observed from the high percentages of “other thermoplastics” (23.4 wt.%), which includes many different materials, and styrenics (39.2 wt.%), formed by ABS and PS. Additionally noteworthy was the high percentage of PVC, which in this case corresponded to electric wires. After passing through the densimetric table, the electrical wires were strongly reduced (from 16.3 to 4.8 wt.%) allowing to obtain 83.7 wt.% of RMSP and 67.2 wt.% of TMR. Hiosta et al. also applied this technique to separate electric wires from WEEE [34]. Dodbiba et al. used the densimetric table to separate PP from PET/PVC fraction and concluded that the densimetric table was effective when the density difference between particles was at least 450 kg m^−3^ [35].

Concerning bulk densities (after being crushed to dp < 8 mm) Table 2 shows that WEEE samples presented the highest values, whereas Film and Paper ones had extremely low densities. That means that Film and Paper samples could present more difficulties when stored, transported or fed to the reactor. The value of the bulk densities of the three samples decreased with the pre-treatments, mainly due to the removal of inorganics. In the WEEE sample, the difference was greater, probably owing to the decrease in the number of wires. In view of Table 2, it can be said that, in general, the pre-treatment techniques employed have proved to be effective for concentrating the plastics, specifically the polyolefins, and reducing PVC, metal and other inorganic materials.

The ultimate and proximate analyses of both raw and pre-treated samples are presented in Table 3 and Table 4, respectively. Table 3 also includes the HHV of the samples. As far as the ultimate analysis is concerned, the percentages of C, H and N corresponded adequately to the composition shown in Table 2. The two samples with the highest plastic content (film and WEEE) showed the highest values of carbon while the sample with high paper content showed the typical carbon values of cellulosic materials. Concerning the H/C ratio, this was in accordance with the nature of the predominant polymers they contain, being the highest for polyolefin-rich samples (Film) and the lowest for styrene plastic-rich samples (WEEE) [18,22]. With regard to nitrogen, the high percentage of this element in the WEEE sample must be noted, which is directly related to its high content of nitrogenous polymers such as ABS, PUR or PA. Finally, it is worth noting the high percentage of chlorine in the Film and WEEE samples, which must mostly come from the PVC they contain. The WEEE sample has a much higher PVC content than the Film sample, and yet both have a chlorine content of around 4 wt.%. The explanation is that, as mentioned above, the PVC counted in the WEEE sample includes electric wires, i.e., it is not only PVC but also copper. After pre-treatment, an increase in C and H is generally appreciated due to the elimination of inorganic materials [36], together with a noticeable decrease in chlorine, related to PVC elimination. This is a very important result in terms of producing pyrolysis oils with low chlorine content. At last, the Film sample showed the highest HHV as a consequence of its high polyolefinic content [37], followed by the WEEE sample and the Paper sample, respectively. In all cases, the pre-treatment techniques caused an increase in HHV, as expected from the elimination of inorganic and low-HHV materials.

Regarding the proximate analysis, all samples were mainly composed of volatile matter, as expected in this type of plastic and paper-rich waste. This is a desirable property because it is from this volatile matter that the pyrolysis oils are formed. Otherwise, it can be seen that the paper samples contained higher moisture and fixed carbon than the rest, as expected from a sample rich in cellulosic material. In addition, the WEEE (raw) sample showed a significant amount of ash, probably coming from the PVC wires and inorganic fillers that may be contained in the plastics of this waste. The ash content was significantly reduced with pre-treatment (also in the other two samples), which increased the amount of volatile matter in the waste, a circumstance that would possibly improve the yield of pyrolysis oils, as mentioned above.

Table 5 shows the metal content of the samples. When analysing this table, the uncertainty associated with the multi-stage analysis of these complex samples must be taken into account. It can be seen that the major metals were calcium, titanium, aluminium and, in the case of the WEEE sample, copper from electric wires. It was surprising that the highest amount of iron was present in the paper sample, as this is an unsuitable material for paper/cardboard waste collection, although it is used in paper and printing ink applications [38]. If such iron came from steel, it is possible that there were no magnetic separators in the waste paper and paperboard sorting plant, and this iron ended up in the rejected fraction under study in this work. Regarding heavy metals (Ni, Pb, Cd, Cr, As, Cu, Co, Tl, Sb, Sn, Hg, Mn, Zn), zinc was the most present, with the exception of copper in the case of the WEEE sample. On this occasion, no clear effect of the pre-treatment could be established for the three samples, although the reduction of the amount of copper in the WEEE sample was evident, which was in agreement with results obtained in previous characterizations.

**Figure 2:**
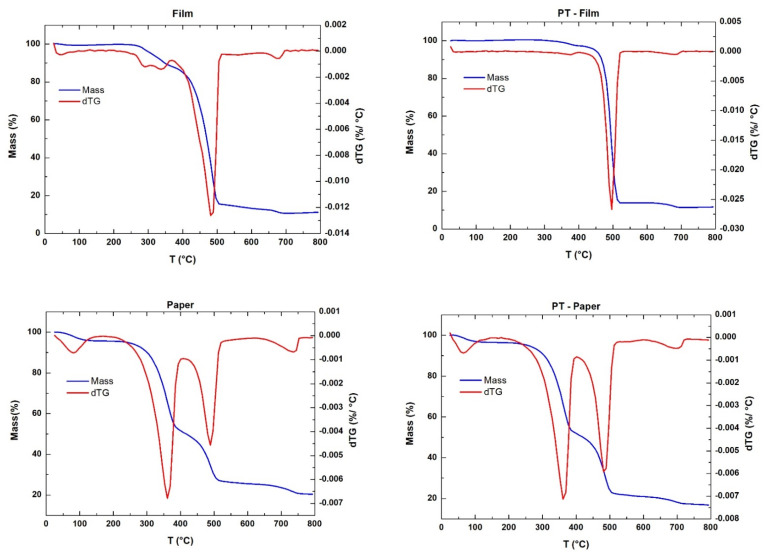

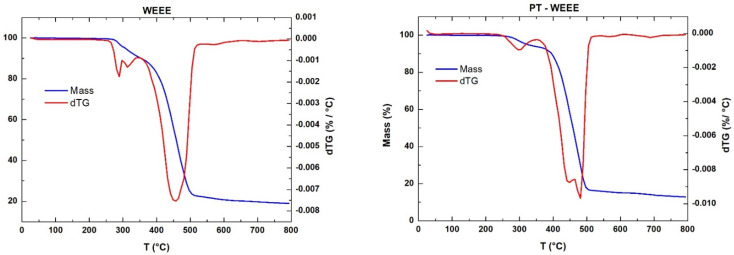
Thermogravimetric profiles of the raw and pre-treated waste samples.

The TGA profiles of all the samples are illustrated in Figure 2, where it can be seen that temperatures slightly higher than 500 °C were needed for the total conversion of the three samples. In view of these results and taking into account that an isotherm of 1 h would be used in the pyrolysis experiments, a lower temperature (460 °C) was selected for the initial experiments, in order to avoid gas formation. As far as the decomposition phenomena occurring in the different samples are concerned, different behaviour can be observed between them. The Film sample showed a decomposition that took place practically in a single step at temperatures close to 500 °C, which is usual in samples whose main content is polyolefins [39]. After pre-treatment, it seemed that decomposition happened in a lower temperature range (narrower DTG peak), which is a consequence of the removal of polymers that can start to decompose at lower temperatures than polyolefins (styrenics, PVC, etc.).

The thermogravimetric profile of the Paper sample showed three main stages of decomposition. (1) The first one close to 100 °C, corresponding to moisture loss, (2) another one around 350 °C, which is related to the decomposition of the cellulosic materials, and the last one (3), at temperatures similar to those observed for the Film sample, corresponding to the cracking of the polyolefins [39]. After pre-treatment, the third DTG peak was higher, as a consequence of the polyolefin concentration resulting from pre-treatment.

Finally, the WEEE sample showed the classical decomposition phenomenon of PVC at 300 °C and the subsequent decomposition of the rest of the plastics, in this case in a wider temperature range than in the case of Film sample, due to the early decomposition of styrene plastics, compared to polyolefins [20]. In fact, in the pre-treated sample, a decoupling at the peak of the main decomposition can be seen, due to the higher percentage of styrenics compared to the raw sample. A smaller peak size can also be observed at 300 °C, due to the lower PVC content.

#### 3.1.2. Pyrolysis Process

The yields obtained in the pyrolysis of the three samples, raw and pre-treated, at 460 °C, are shown in Table 6. Regarding the film-rich samples (raw and pre-treated), it can be seen that pyrolysis oils were the main product, followed by gas and solid. These results are directly attributed to the high polyolefin content in the initial sample (Table 2). In particular, the liquid yield of the pre-treated film sample reached 70.6 wt.% owing to the 93.0% of RMSP present in the feedstock. These results are in accordance with those obtained in previous papers. Lopez et al. obtained 65 wt.% of liquid yield at 500 °C using a sample that contained 92.3 wt.% of plastic [5]. Yan et al. reported the pyrolysis of PP and LDPE waste at 460 °C, reaching the 65.4 wt.% and 77.1 wt.% liquid yields, respectively [40]. Regarding the effect of pre-treatment, the increase in the yield of liquids can be related to the decrease in the yield of solids. Such a decrease in solid yield can be explained by the elimination of inorganic compounds and polymers that have a tendency to carbonize (PVC, PET, cellulose) during the pre-treatment.

In the case of the Paper sample, the main fraction was the solid one, followed by liquids and gases. Such performance in solids can be explained by the high amount of inorganics contained in this sample (13.4 wt.%), together with a large amount of polymers with a tendency to carbonize, mainly cellulosic materials (31.5 wt.%). It is remarkable that this sample produced an aqueous liquid phase. This is explained by the presence of cellulosic-based materials rich in -OH and =O groups [5,20]. In the case of this sample, pre-treatment reduced the total solid yield by half and increased more than twice the organic liquid yield (from 17.8 to 42.5 wt.%). This fact might be explained by the significant effect of the pre-treatment on the reduction of (1) inorganic content (13.4 to 2.5 wt.%), and (2) char precursor materials, PET (5.3 to 2.7 wt.%) and especially cellulose (from 31.5 to 18.3 wt.%). However, the aqueous liquid phase could not be completely removed by the pre-treatment.

Finally, the WEEE sample generated also liquids as the main product, followed by solids and gases. Concerning the high solid yield, this sample did not contain a lot of inorganic material as such (2.3 wt.%), but it is necessary to remember the aforementioned issue of electric wires; in fact, the ash content determined by proximate analysis was high (22.0 wt.%, Table 4). Furthermore, the group constituted by “other thermoplastics” contained polymers with a tendency to carbonize and within “multimaterial” there were some inorganic elements coming from circuit printed boards. After pre-treatment, the higher liquid yield was observed and, at the same time, the solid yield decreased, as a consequence of the removal of PVC, inorganics and multimaterial. The liquid yield obtained from these two WEEE samples was similar to those obtained by Caballero et al. when investigating the pyrolysis of WEEE plastics at 500 °C. They found that landline waste (phones) generated a 58 wt.% liquid yield while mobile phones a 54 wt.% [22]. Higher values (around 70 wt.%) were obtained by Hall et al. during pyrolysis of mixed WEEE in a fixed bed reactor at 600 °C [41].

To summarize, it can be said that for the three different waste samples, the pre-treatment led to higher liquid yields and lower solid yields as compared to the pyrolysis of raw samples, while gas yields remained almost constant. This is the evidence that the pre-treatments produced the desired effect, which is the promotion of pyrolysis oils through the elimination of undesired materials of the original samples.

#### 3.1.3. Pyrolysis Oils

Liquid products had a different appearance depending on the composition of the pyrolyzed waste sample. The liquids obtained from Film waste samples resulted in a waxy-like product instead of liquid oil, which can be ascribed to the high H/C ratio of the waste samples (see Table 3), principally explained by their great PE content [42,43]. In fact, Kiran et al. and Sharudin et al. experimented with operational blockage problems in pipelines and condenser tubes with waxes formation when pyrolyzed samples richer in PE [44,45]. Nevertheless, these waxes obtained from the polyolefins pyrolysis can serve as feedstock for FCC units of petroleum refineries [46,47]. Paper samples presented two differenced phases (organic and aqueous) in the liquid as was explained in Section 3.1.2. The organic phase of the pre-treated paper oil presented a more waxy-like appearance than the non-pre-treated one according to the promotion of polyolefins with the pre-treatment (see Table 2). By contrast, the pyrolysis of WEEE samples, with an aromatic/naphthenic nature, result in dark-brown coloured oils, which resemble petroleum fractions [5,18,22].

In order to evaluate the quality of the organic liquid products, several of their properties, such as higher heating value (HHV), halogen and metal content, and composition, were determined. First, the most limiting properties, i.e., HHV, halogen content and metal content, were analysed. This information is presented in Table 7 and Table 8. Table 7 shows the HHV and the halogen content of these liquid products. The HHV of the pyrolysis oils was high (40–43 MJ kg^−1^) and close to those of liquid fossil fuels (diesel 45 MJ kg^−1^ and heavy fuel oil 42–43 MJ kg^−1^ [43]), with the exception of Paper samples (37–39 MJ kg^−1^). This is an important result, as it provides the possibility of using these oils as alternative fuels. Again, the pre-treatment improved the calorific properties of the pyrolysis oils, increasing the HHV in all cases due to the reduction of impurities and PET [36], and the concentration of MSP.

On the other hand, it is important to consider the halogen content, since they have an important and negative impact on the direct application of pyrolysis oils as fuels [20,48]. In this work, fluorine, chlorine and bromine were measured. It is clear from Table 7 that the main halogen element in the pyrolysis oils was chlorine. The fluorine and bromine values were very low, with the exception of the bromine content of the liquids from the WEEE samples, probably due to the presence of brominated flame retardants as part of the additives in the plastic materials of this waste. Liquids generated from raw waste samples presented more chlorine content, directly related to the presence of PVC, as was concluded by several authors [20,49,50]. Although all pre-treatments achieved a reduction in the halogen content, pyrolysis liquids still showed relatively high chlorine content. PT-Film sample registered the lowest chlorine content (160 ppm). This value, although low, is higher than the value established for use in existing petrochemical plants (3–10 ppm), as stated by some authors [51,52,53] However, these pyrolysis oils could probably be blended with other refinery streams before usage and most likely could be used as alternative fuels in cement kilns, where the required chlorine concentration is not usually so low. In the conditions of this work, about 10–30 wt.% of the chlorine content present in the waste samples was transferred to the liquid product. These transfer ratios can be reduced in several ways: using solid catalysts or adsorbents [20,49,54] or by the application of stepwise pyrolysis (two-stage pyrolysis) [55] but this was out of the scope of this paper.

Table 8 shows the metal content of the pyrolysis oils. In this case, two issues need to be considered: the heavy metal content, which could lead to environmental problems, and the presence of metals that could act as poisons in catalysts used in petrochemical processes. With regard to heavy metals (in bold in the table), zinc, antimony, lead, nickel, manganese and chromium were detected, all in concentrations below 8 ppm in the oils from the pre-treated samples. This means that all these oils were free of heavy metals such as cadmium, copper, arsenic, cobalt, thallium, tin and mercury, or at least the concentration of these metals was below 1 ppm. Among the metals that can cause problems in catalysts, the presence of silicon was particularly noticeable in the oils from the WEEE samples. For this reason, it is important to take it into account when processing oil in the refinery, since requirements are usually established to avoid its presence and prevent damage to the catalysts. The limits of the metals will depend on each refinery, the processing unit in which the oil is included and the degree of dissolution that the oil presents along with the conventional feed used.

**Figure 3:**
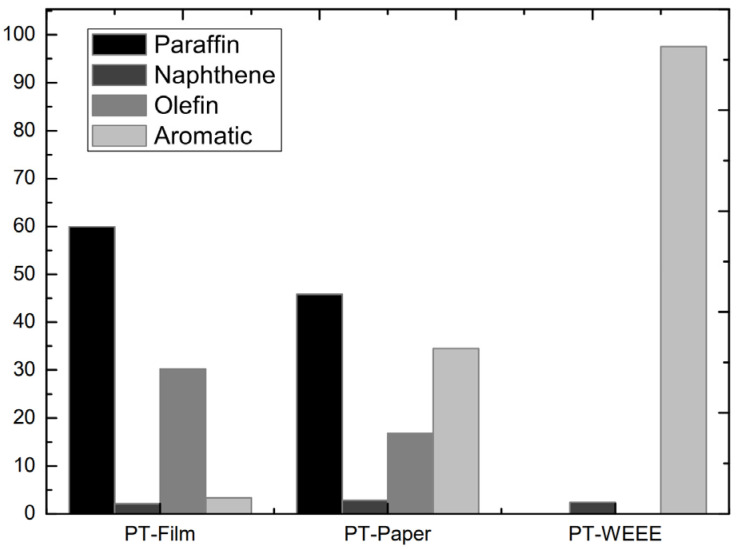
Composition of pyrolysis oils coming from pre-treated samples.

As the liquid fractions coming from the pre-treated samples showed better quality, the characterization by the GC/MS was carried out only in these oils. Figure 3 shows the compounds identified by this method grouped according to their nature in paraffinic, naphthenic, olefinic and aromatic compounds. Only those compounds with areas > 1% and an identification quality degree > 90% were included in such groups. The oil from the pre-treated Film sample was composed mainly of paraffins (59.9% area) and olefins (30.2% area), due to the high content of PE presented in the original sample (see Table 2). It was proven by other authors that during the degradation of PE, free radical fragments are formed and react with hydrogen chains, giving rise to alkanes and alkenes [5,56]. According to Das et al., olefins are the precursors of many industrial organic chemicals such as vinyl acetate, acetaldehyde and vinyl chloride, therefore, the concentration of olefins in the pyrolytic oil could be used in numerous industrial applications [57].

On the other hand, pre-treated WEEE sample oil consisted of more than 97% area of aromatic compounds, with small quantities of naphthenes (2.4% area). The high quantity of aromatics is attributed to the great styrene content and the low content of polyolefins in the original sample (see Table 2). In previous investigations, 80% of aromatic hydrocarbons were obtained in the pyrolysis of PS [58]. Since a high concentration of aromatics is desired for gasoline production [10], this could be the most appropriate application for PT-WEEE oil provided the chlorine content is reduced. The major compounds in the PT-Paper sample oil included paraffins (45.8% area), naphthenes (16.9% area) and aromatics (34.5% area). This wide distribution is related to the composition of the sample. As was previously mentioned, polyolefins generate paraffinic and olefinic compounds while styrenics favour aromatic content in the liquid oils. Moreover, other fractions such as PET could also favour the formation of the former compounds [15].

### 3.2. Effect of Temperature in the Production of Oils Coming from PT-Film Sample

At this point of the investigation, it was considered that the PT-Film sample was the most suitable sample to deepen the possibilities of pyrolysis oil production. This decision was based on the fact that the composition of these liquids allowed them to be considered a priori as feedstock for refineries or as a source of olefins, and the HHV and halogen content enabled its use as an alternative fuel. In addition, it was the sample that generated the greatest amount of liquids. Therefore, this sample was selected to study the effect of the cracking temperature, which is the most significant variable in the pyrolysis process, showing a critical influence in the conversion and product distribution [59]. The pyrolysis experiments were run at three different temperatures: 430, 460 and 490 °C. The yields are presented in Table 9.

As it can be seen in Table 9, solid and liquid yields were strongly affected by temperature, while the gas formation did not show such a wide variation. The liquid was the main product and its yield rose with the increase in temperature from 48.8 wt.% at 430 °C to 78.0 wt.% at 490 °C. Equally, an important decrease in solid yield was observed in the same temperature range (from 38.7 to 7.8 wt.%). This fact indicates that pyrolysis was incomplete until 490 °C, that is, organic matter was still remained for cracking in the experiments carried out at lower temperatures. This phenomenon was previously reported in pyrolysis tests carried out at temperatures below 500 °C with similar samples [16]. As far as gas yield is concerned, the most common thing is to observe a trend of higher gas yields as the temperature increases, due to the stronger breaking of the polymer chains that happens at high temperatures, as happened in this work [28,48].

The temperature effect was also investigated in the properties of pyrolysis oils. HHV and halogen content are presented in Table 10. Concerning HHV, a slight increase in the HHV was produced as the temperature rose, ranging 44.3 MJ kg^−1^ at 490 °C (Heavy fuel oil: 42–43 MJ kg^−1^). The same tendency was found in other works [36]. As was mentioned before, halogen content, especially chlorine, is limited by the requirements of refineries. In general, no significant effect of the temperature on the halogen content was found. Concerning chlorine content, higher temperatures led to a slightly lower presence of chlorine in the liquid products. However, previously published papers concluded that there is usually a chlorine increase with temperature (from 460 °C to 600 °C) as a result of the quicker interactions between radical fragments and HCl released from PVC [16]. Anyway, this depends to a large extent on the operating conditions and the design of the pyrolysis plant. Moreover, the differences in this work were not very significant (units are in ppm), and could be part of the intrinsic error of experimentation and analytics.

**Figure 4:**
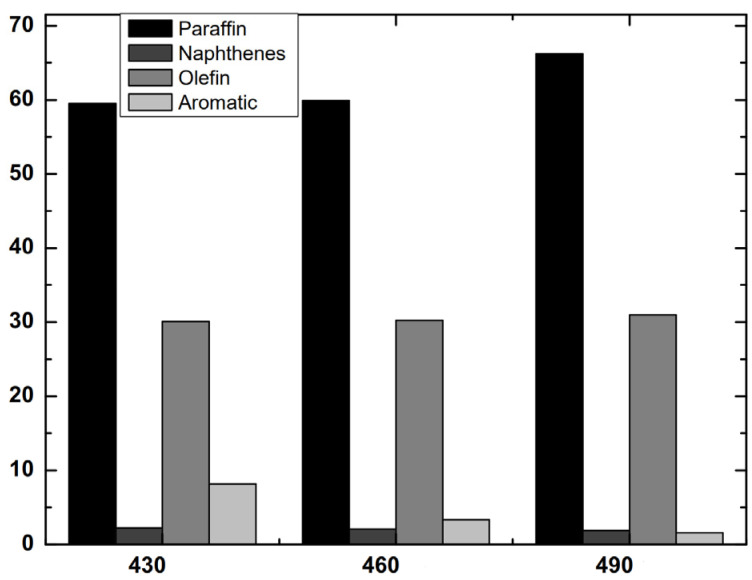
Composition of pyrolysis oils of PT-Film pyrolyzed at different temperatures.

Concerning composition, in spite of the increase in the cracking temperature, all liquids were wax-like products that solidified at ambient temperature and easily re-melted above 40 °C. Nevertheless, it was reported that higher cracking temperatures can decrease the viscosity of the liquids. This effect is observed at operating temperatures above 600 °C when waxes chains are broken down to lighter components due to the higher thermal cracking produced [15,27]. However, there is no evidence of this effect at the temperature range studied in this research. Figure 4 shows the distribution of the hydrocarbon’s nature from the compounds identified by GC/MS. It was discussed in Section 3.1.3 that the oil coming from PT-Film mainly consisted of paraffinic and olefinic compounds due to the original composition of the sample. Now, as the temperature increased, the paraffin content raised whereas aromatic distribution was reduced. This is due to the fact that the temperature favours the intramolecular hydrogen transfer, generating a more paraffinic fraction [57,60,61]. Nevertheless, other authors experimented with the reverse trend, Onwudili et al., who pyrolyzed LDPE and PS in a batch reactor from 300 °C to 500 °C, concluded that higher temperatures and higher residence times favoured the aromatic proportion in pyrolytic oils due to the cyclization and aromatization at 500 °C [62] The explanation for the difference between these results can lie in the different designs of reactors and reaction systems, which have relevant importance in the routes and mechanisms of reaction that take place. In any case, the removal of aromatics in these pyrolysis oils is a good result, as this means a purer stream of paraffins and olefins.

**Figure 5:**
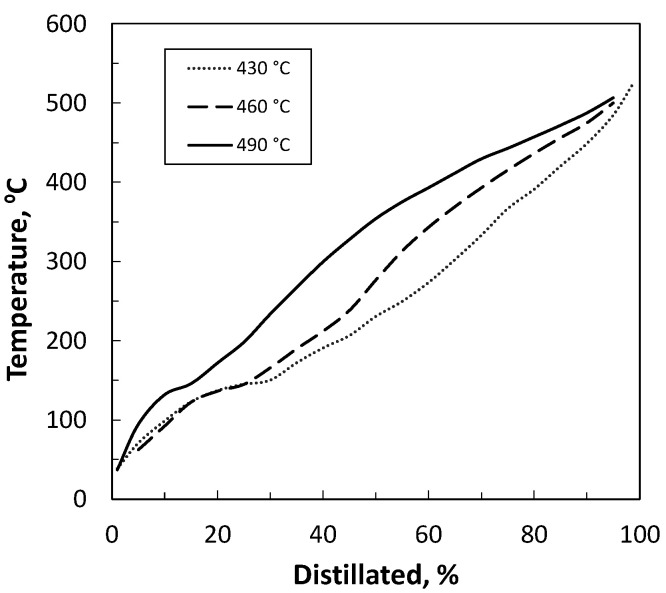
Simulated distillation curves of pyrolysis oils of PT-Film pyrolyzed at different temperatures.

At last, the results of simulated distillation are presented in Figure 5 and Table 11. The raw distillation curve (presented in Figure 5) showed that the final boiling point at T95% was 506.6 °C for the oil obtained at 490 °C, 500 °C for the oil obtained at 460 °C and 485 °C for the oil obtained at 430 °C. Moreover, the hydrocarbon fractions were classified based on their boiling temperature: naphtha (T < 216 °C), middle distillates (216 °C < T < 343 °C) and heavy diesel (T > 343 °C). Attending to the results shown in Table 11, a temperature effect can be observed: rising temperature reduced the light fraction (naphtha) while the heavy fraction (heavy diesel) was increased. Other authors reported the same effect [16,60].

## 4. Conclusions

Pyrolysis appears as an attractive alternative for recycling rejected streams with high plastic content. The idea is to obtain pyrolysis oils that can be used for petrochemical processes or as alternative fuels. However, industrial rejected streams present different natures depending on their origin and this decisively influences the production of pyrolysis oils. After analysing rejected streams from sorting plants for packaging waste (Film sample), paper/cardboard waste (Paper sample) and waste from the electrical and electronics sector (WEEE sample), it was found that they contain significant quantities of materials that can reduce the quantity and quality of pyrolysis oils. These materials are mainly PVC, PET and cellulosic materials, and inorganic matter such as metals, which lead to the generation of chlorinated oils (PVC), aqueous phases in the oils (PET and cellulosic materials) and high quantities of pyrolysis solids in detriment of liquids (inorganic matter as metals).

These samples were subjected to mechanical separation processes (pre-treatments) and all pre-treatments were effective in concentrating materials suitable for pyrolysis (mainly polyolefins and styrenic plastics). Flotation and densimetric separation achieved a high recovery rate for the Film and WEEE samples, respectively. By contrast, a great deal of material mixture in the Paper sample made the separation by NIR spectroscopy less effective. Nevertheless, all the pre-treated samples achieved higher liquid and lower solid yields compared with raw samples. Regarding the quality of pyrolysis oils, the higher heating value of the oils coming from pre-treated Film and WEEE samples were similar to heavy fuel oil, showing its potential application as fuel. Moreover, the oil from the pre-treated Film sample was mainly composed of olefins and paraffins, whereas the oil coming from the pre-treated WEEE sample was based on aromatic compounds. The halogen content was considerably reduced in the oils after pre-treatment; however, a significant proportion of chlorine was transferred to oils, limiting its application.

The temperature effect was also studied in the 430–490 °C range, using the pre-treated Film sample. The temperature favoured the formation of liquid products (from 48.8 to 78.0 wt.%) and solid yield decreased (from 38.7 to 7.8 wt.%). In addition to increasing liquid production, at 490 °C, an oil with very low chlorine concentration (128 ppm), high HHV (44.3 MJ kg^−1^) and high paraffin content was produced. The results presented in this work demonstrate that the implementation of mechanical material separation processes can be an interesting option as a preliminary step to pyrolysis processes, with the aim of producing more quantities of pyrolysis oils with improved properties. This information should be taken into account when designing recycling processes for complex waste by pyrolysis.

## Figures and Tables

**Table 1:** Annual production, industrial activity and aspect of the three samples used.

Sample	Film	Paper	WEEE
Annual production (t/year)	3491	24,341	13,228
Activity	Separation of light packaging	Recycling of paper	WEEE treatment
Aspect	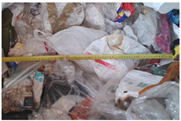	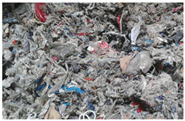	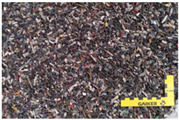

**Table 2:** Material composition (wt.%) and bulk density (kg m^−3^) of raw and pre-treated samples.

Material	Film Sample		Paper Sample		WEEE Sample	
	Raw	Pre-Treated	Raw	Pre-Treated	Raw	Pre-Treated
Polyolefins (PP, PE)	75.0	93.1	36.1	68.0	14.6	19.0
Styrenics (PS, ABS)	1.7	1.0	8.8	3.6	39.2	47.9
PVC	4.5	0.0	2.8	1.5	16.3 ^2^	4.8 ^2^
PET	3.4	0.0	5.3	2.7	1.4	1.0
Other thermoplastics ^1^	0.1	0.0	0.0	0.0	23.4	23.4
Multimaterial	3.4	4.5	1.2	1.4	2.3 ^3^	1.3 ^3^
Other organic	0.8	1.1	0.0	0.0	0.0	0.0
Inorganic matter	5.4	0.0	13.4	2.5	2.3	1.4
Celluloses	5.5	0.1	31.5	18.3	0.5	1.2
Textile	0.2	0.2	0.9	2.0	0.0	0.0
Bulk density	0.093	0.036	0.253	0.050	1.620	0.510
TMR	-	78.5	-	27.4	-	67.2
RMSP	-	93.0	-	43.7	-	83.7

^1^ PMMA, PUR, PC, PA, PBT, POM; ^2^ Including electric wires; ^3^ PCB + rubber.

**Table 3:** Ultimate analysis (wt.%) and HHV (MJ kg^−1^) of raw and pre-treated waste samples (as received).

Sample	C	H	N	S	Cl	H/C	HHV
Film	70.5	11.2	0.4	<0.1	4.1	1.91	36.3
PT-Film	75.6	12.3	0.5	n.d. ^1^	0.2	1.95	38.0
Paper	46.8	6.8	0.3	0.2	1.6	1.74	22.8
PT-Paper	55.9	8.2	0.4	n.d. ^1^	0.9	1.76	27.0
WEEE	64.4	7.0	1.2	<0.1	4.4	1.30	26.9
PT-WEEE	74.7	7.8	1.9	n.d.^1^	1.2	1.25	33.9

^1^ Not determined.

**Table 4:** Proximate analysis of raw and pre-treated waste samples (wt.%, as received).

Sample	Moisture	Volatile Matter	Fixed Carbon ^1^	Ash
Film	0.7	91.1	1.6	6.6
PT-Film	0.3	93.1	0.4	6.2
Paper	3.5	77.9	7.0	11.6
PT-Paper	2.4	82.9	5.3	9.4
WEEE	0.0	76.6	1.4	22.0
PT-WEEE	0.0	88.7	2.4	8.9

^1^ By difference.

**Table 5:** Metal content in raw and pre-treated samples (ppm, as received).

Metal	Film	PT-Film	Paper	PT-Paper	WEEE	PT-WEEE
Zn	86.5	107	114	72	457	222
Sb	7.7	3.1	7.7	5.0	<1	621
P	169	133	60.4	236	269	818
Pb	<1	7.2	8.6	3.5	<1	91.1
Co	<1	4.5	3.2	38.8	9.3	7.9
Cd	<1	<1	<1	<1	10.6	22.4
Ni	<1	< 1	20.2	8.3	65.5	58.7
Fe	399	323	4257	949	498	632
B	<1	<1	<1	<1	33.3	16.4
Si	175	118	320	500	270	246
Mn	10.2	9.1	31.7	18.5	115	174
Cr	3.8	4.1	32.2	6.4	16.3	15.1
Mg	182	183	559	334	763	550
Ca	14,620	13,890	12,060	17,390	13,820	8022
Cu	22.0	32.4	76.5	24.7	48620	6337
Ti	6546	8173	1520	2993	5842	6264
Al	8463	3620	17430	6960	25,580	27,820
Na	253	192	843	421	75	68.8

Concentration of Sn, Tl, As, Mo, Ba, V and Ag was <1 ppm.

**Table 6:** Pyrolysis yields of the raw and pre-treated samples (wt.%).

Sample	Oils		Gas ^1^	Solid
	Organic	Aqueous		
Film	61.0	0.0	14.3	24.7
PT-Film	70.6	0.0	12.8	16.6
Paper	17.8	19.9	21.7	40.6
PT-Paper	42.5	10.9	20.0	26.6
WEEE	51.6	0.0	19.9	28.5
PT-WEEE	60.1	0.0	20.8	19.1

^1^ By difference.

**Table 7:** HHV (MJ kg^−1^) and halogen content (ppm) of the organic fraction of pyrolysis oils.

Sample	HHV	F^−^	Cl^−^	Br^−^	% Cl^−^ Transferred
Film	40.4	57	12,213	13	30
PT-Film	42.6	27	160	<10	8
Paper	37.4	26	1479	42	9
PT-Paper	39.2	7	894	11	10
WEEE	39.7	19	13,078	709	30
PT-WEEE	40.3	17	2076	796	17

**Table 8:** Metal content (ppm) in the pyrolysis oils from raw and pre-treated samples.

Metal	Film	PT-Film	Paper	PT-Paper	WEEE	PT-WEEE
Zn	8.3	5.9	8.2	6.0	<1	<1
Sb	7.7	3.1	7.7	5.0	<1	<1
P	5.5	<1	< 1	2.8	92.7	95.8
Pb	5.1	7.0	< 1	7.1	6.5	6.2
Ni	6.9	10.0	5.5	3.1	<1	<1
Fe	41.3	47.0	30.9	12.0	16.0	<1
Si	106	290	876	217	1813	567
Mn	<1	2.0	<1	<1	<1	<1
Cr	11.1	7.7	7.4	3.4	2.5	<1
Mg	<10	228	<10	<10	<10	<10
Ca	53.5	319	100	68.9	59.4	50.2
Al	21.6	4.2	6.1	4.8	5.8	3.4
Na	<10	70.8	<10	<10	20.6	<10

Concentration of Co, Cd, Cu, Sn, B, Tl, Ti, As, Mo, Ba, V and Ag was <1 ppm.

**Table 9:** Pyrolysis yields of PT-Film sample at different temperatures (wt.%).

Temperature (°C)	Oils		Gas ^1^	Solid
	Organic	Aqueous		
430	48.8	0.0	12.5	38.7
460	70.6	0.0	12.8	16.6
490	78.0	0.0	14.2	7.8

^1^ By difference.

**Table 10:** HHV (MJ kg^−1^) and halogen content (ppm) of the pyrolysis oils of PT-Film pyrolyzed at different temperatures.

Temperature (°C)	HHV	F^−^	Cl^−^	Br^−^
430	42.2	14	245	<10
460	42.6	27	160	<10
490	44.3	12	128	<10

**Table 11:** Distillation fractions of pyrolysis oils of PT-Film pyrolyzed at different temperatures.

Temperature (°C)	Naphtha	Middle Distillates	Heavy Diesel
430	45.8	25.4	28.8
460	39.9	20.0	40.1
490	26.3	21.8	51.9
